# Description of the geographic distribution of excessive drinking across regional cultures in the United States: Framing an important health metric according to the cultural context of the American nations

**DOI:** 10.1371/journal.pone.0344249

**Published:** 2026-03-04

**Authors:** Shane A. Phillips, Ross Arena, Nicolaas P. Pronk, Colin Woodard

**Affiliations:** 1 Department of Physical Therapy, University of Illinois, Chicago, Illinois, United States of America; 2 Healthy Living for Pandemic Event Protection (HL – PIVOT) Network, Chicago, Illinois, United States of America; 3 HealthPartners Institute, Minneapolis, Minnesota, United States of America; 4 Department of Health Policy and Management, University of Minnesota, Minneapolis, Minnesota, United States of America; 5 Nationhood Lab, Pell Center for International Relations and Public Policy, Salve Regina University, Newport, Rhode Island, United States of America; University of Modena and Reggio Emilia: Universita degli Studi di Modena e Reggio Emilia, ITALY

## Abstract

Excessive alcohol drinking results in an increased risk for chronic diseases, and the rates of alcohol consumption have increased among U.S. adults in the past decade. The U.S. Surgeon General called for updating consumer labels to include this risk. This paper aims to understand the regional distribution of excessive drinking and how these patterns may be explained according to the *American Nations* model of the first U.S. settlement streams. We present data from the 2024 County Health Rankings program to demonstrate the distribution of excessive drinking, showing that excessive alcohol drinking patterns are region-specific and predicted by the *American Nations* model. This paper introduces the *American Nations* model in promoting alcohol consumption reduction messages.

## Introduction

The United States (U.S.) populace has high levels of physical inactivity, obesity, poor nutrition, excessive drinking patterns, and other chronic health conditions and unhealthy living characteristics. Excessive drinking – which includes binge drinking (>4 drinks in women; > 5 drinks in men in a 2-hour period) and heavy alcohol use (≥5 drinks in a day, ≥ 15 in a week in men; ≥ 4 drinks in a day, ≥ 8 drinks in the past week in women) -- results in a significant risk for disease. These patterns of drinking were on a downward trend from 2011 to 2018, but over the last decade, have increased among U.S. adults [1]. In 2018 it was estimated that 1 in 6 adults binge drink and that there were 178,000 deaths attributed to excessive drinking per year, making it a leading cause of preventable death [2]. The commonly accepted health risks associated with binge and excessive drinking include high-risk sexual behavior, physical injury, and motor vehicle crashes [3]. Several retrospective studies of adults aged 40–60 years indicated that binge drinking was associated with a heightened risk of cardiovascular (CV) events, including stroke and myocardial infarction [4]. For example, analyzing data from the U.S. National Health and Nutrition Examination Survey, we established that young men who report repeated excessive alcohol consumption have higher systolic blood pressures (BP) compared to young men who don’t drink excessively [5].

Healthcare professionals need new approaches to help adults understand the risks of and reduce excessive drinking and binge drinking behaviors. Existing binge drinking reduction approaches include brief intervention, personalized feedback, personalized normative feedback, and/or interventions that modify alcohol outcome-expectancies [6,7]. These interventions have been delivered via in-person groups and in face-to-face settings such as emergency room departments and have been successful in lowering alcohol consumption in some communities, though the impact has not been universal [8]. These unequivocal findings suggest that new approaches need to be considered to study healthy living characteristics with the cultural and regional distribution of drinking patterns.

Recently, this group has found that there is a heterogenous geographic distribution of unhealthy living characteristics and various types of chronic disease in the U.S., with clear *hot spots* across the country [9,10]. Further, these *hot spots* appear to co-exist with other co-morbidities and poor health outcomes, such as COVID-19 mortality, in areas that have the highest prevalence of unhealthy living and chronic disease metrics. In a recent analysis, it was found that deaths related to excessive drinking quadrupled in the first year of the COVID-19 pandemic compared to the previous 2-year period [2]. Further potential contributing factors include greater lockdown-era access to alcohol (through increased sales options) and reduced availability of emergency medical services [11]. On the other hand, the identification of regional differences in the prevalence of co-morbidities and healthy living characteristics, which may also play a role in alcohol-related disease and mortality, deserves further attention.

As mentioned, strategies to reduce excessive drinking have yielded mixed results [3]. Some of these approaches have shown positive effects on drinking frequency but reported effect sizes were small or indicated little change in drinking behaviors, suggesting the need to develop and test other approaches [6]. Alternate approaches have shown some promise, with alcohol tax and levels having been shown to be inversely related to excessive drinking and other alcohol-related health outcomes.^32^ However, taxation tends to be regionally distributed across the U.S. and may not be universally accepted by people and alcohol beverage-producing industries in certain regions. The American Nations model of U.S. regional cultures was utilized in this study to help understand how excessive drinking prevalence may vary by U.S. region and geography.

The purpose of this report is to understand the regional distribution of excessive drinking patterns in the U.S. and how these patterns may be explained according to the *American Nations* model of settlement streams. Importantly, given the disparities in outcomes associated with public health messaging and alcohol health policy, it may be more effective to optimize messages and interventions by tailoring the approach to the unique cultural characteristics and values of a given geographical location.

## Materials and methods

County-level excessive drinking data was obtained from the 2024 County Health Rankings (CHR) program of the University of Wisconsin Population Health Institute [12]. Excessive drinking was reported as an age-adjusted percentage and defined as “the percentage of adults that report binge or heavy drinking in the past 30 days.” [13] Source data for excessive drinking came from the Behavioral Risk Factor Surveillance System (BRFSS) [14].

The *American Nations* regional model was obtained from the Nationhood Lab [15]. A brief description of the American Nations model is as follows: Cultural geographers have long recognized First Settler effects on the characteristics of national cultures, with Wilbur Zelinsky’s “Doctrine of First Effective Settlement” [16] arguing that “the dominant culture of a given nation is determined by the characteristics of the first group of settlers regardless of how small the initial band of settlers might have been.” Regional cultures can be discerned and mapped by tracking competing first settlement streams, an exercise that has informed the work of historians [17,18] and geographers [19–21]. This American Nations model [22] has been applied to explain differences in areas such as economic development, [23] mortality [24], gender wage gaps [25], and health characteristics [9] such as diabetes and obesity [9] and other healthy living characteristics such as physical inactivity, unhealthy dietary patterns, and sleep [10,26]^,31^. A detailed description of the *American Nations* cultures has been previously published ^9,24^. Descriptions of the unique cultural characteristics of the American Nations are summarized here again in Table 1 ^31^.

**Table 1:** Brief descriptions of the identities of the ‘American nations’.

1	Yankeedom Founded by Puritans who sought to perfect earthly society through social engineering, individual denial for common good, and the assimilation of outsiders. The common good – ensured by popular government – takes precedence over individual liberty when the two are in conflict.
**2**	**New Netherland** Dutch-founded and retains characteristics of 17th century Amsterdam: a global commercial trading culture, materialistic, multicultural, and committed to tolerance and the freedom of inquiry and conscience.
**3**	**Tidewater** Founded by lesser sons of landed gentry seeking to recreate the semi-feudal manorial society of English countryside. Conservative with strong respect for authority and tradition, this culture is rapidly eroding because of its small physical size and the massive federal presence around D.C. and Hampton Roads.
**4**	**Greater Appalachia** Original settlers overwhelmingly from war-ravaged Northern Ireland, Northern England and Scottish Lowlands and were deeply committed to personal sovereignty and intensely suspicious of external authority.
**5**	**The Midlands** Founded by English Quakers, who believed in humans’ inherent goodness and welcomed people of many nations and creeds. Pluralistic and organized around the middle class; ethnic and ideological purity never a priority; government seen as an unwelcome intrusion.
**6**	**Deep South** Established by English Barbadian slave lords who championed classical republicanism modeled on slave states of the ancient world, where democracy was the privilege of the few and subjugation and enslavement the natural lot of the many.
**7**	**New France** An enclave of a larger culture encompassing Quebec and parts of Atlantic Canada, the legacy culture was consensus driven, tolerant, and comfortable with government involvement in the economy, though these characteristics appear to have collapsed in much of Cajun country in recent decades.
**8**	**El Norte** Borderlands of Spanish-American empire, so far from Mexico City and Madrid that it developed its own characteristics: independent, self-sufficient, adaptable, and work-centered. Often sought to break away from Mexico to become independent buffer state, annexed into U.S. instead.
**9**	**Left Coast** Founded by New Englanders (who came by ship) and farmers, prospectors and fur traders from the lower Midwest (by wagon), it’s a fecund hybrid of Yankee utopianism and the Appalachian emphasis on self-expression and exploration.
**10**	**Far West** Extreme environment stopped eastern cultures in their path, so settlement largely controlled by distant corporations or federal government via deployment of railroads, dams, irrigation, and mines; exploited as an internal colony, with lasting resentments.
**11**	**First Nation** Populated by indigenous groups that generally never gave up their land by treaty and have largely retained cultural practices and knowledge that allow them to survive in this hostile region on their own terms. This nation is now reclaiming its sovereignty, having won considerable autonomy in Alaska and Nunavut and a self-governing nation state in Greenland that stands on the threshold of full independence.
12	**Greater Polynesia** An enclave (in the state of Hawaii) of the massive Western Pacific cultural space settled by the great celestial navigators. Communitarian social structure. Extends thousands of miles across French Polynesia, the Cook Islands, Samoa and Tonga.
13	**Spanish Caribbean** The northern tip of Spain’s maritime cultural space in the Caribbean basin, a distinct culture from El Norte with an epicenter in Havana and including today’s Puerto Rico and Dominican Republic.

Note: Reprinted with permission from: Pronk NP, Arena R, Laddu D, Woodard C. Regional cultures and insufficient sleep in the United States. *Journal of Activity, Sedentary, and Sleep Behaviors*. 2024;3:4. https://doi.org/10.1186/s44167-023-00043-3

The CHR and Nationhood Lab databases both contained U.S. Federal Information Processing Series (FIPS)-code identifiers. As such, the databases used in the current study were linked through FIPS-code identifiers using Microsoft Excel (Redmond, WA). HealthPartners Institute Research Subjects Protection Program determined that this study is exempt from IRB review and ongoing oversight under 45 CFR Part 46 as it involves the analysis of existing, publicly available data.

## Results

**Fig 1:**
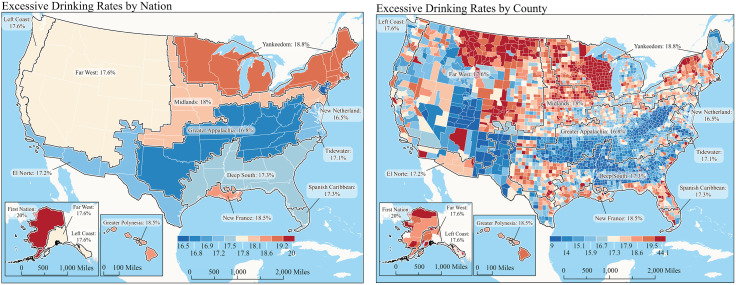
Prevalence of excessive alcohol consumption. Panel 1a represents excessive drinking rates by nation as an expression of percentile. Panel 1b represents excessive drinking rates by county. Maps are produced by the authors at the Nationhood Lab. https://www.nationhoodlab.org/.

**Fig 1 (a and b)** illustrate the 2024 prevalence of excessive drinking in the American Nations, both as an overall prevalence in each region and at the county level. Heterogeneity in excessive drinking prevalence is observed, and there are several apparent excessive drinking hot spots across the northern regions that encompass several of the *American Nations*, including Yankeedom, Midlands, New France, First Nation, and Greater Polynesia, exceeding 18%. Focal points of higher prevalence within these belts exist within the First Nation and Yankeedom regions. The pattern of excessive drinking prevalence illustrated in Fig 1a and 1b shows overlapping similarities to the lower patterns of physical inactivity and obesity that were previously reported in these regions.^9^ On the other hand, these studies found that the regions of higher prevalence of physical inactivity and obesity were Appalachia and the Deep South (obesity prevalence: 36%; physical inactivity: 25–27%), while areas of lower physical inactivity and obesity were Midlands and Yankeedom [9]. The regions with the higher prevalence of excessive drinking appear to coexist with previous prevalence studies by this group, showing the higher rates of food insecurity and limited access to healthy foods in these same regions [10].

The reference map (Fig 1a and 1b) represents a novel graphic of excessive drinking patterns across geographic areas. According to the American Nations model, this approach may offer helpful insights into patterns of excessive drinking, which may be actionable by region-specific interventions. Using this model, the prevalence of excessive drinking, as defined by the BRFSS data, suggests regional heterogeneity.

## Discussion

Public health policy regarding alcohol consumption has shown some promise in reducing drinking patterns, such that population-level alcohol pricing policies have been found to reduce consumption by 7–8% for every 10% increase in the minimum price of alcohol [27]. In Canada, this type of policy shift towards higher pricing and taxation of alcohol has also been associated with reductions in health-related harm, such as traffic fatalities and alcohol-associated disease mortality [28]. However, the long-term impact of such a policy shift in the U.S., as it relates to CV disease, cancer, and other chronic diseases, is unknown. Further, the political feasibility of addressing alcohol consumption patterns in the U.S. is likely to vary by state and by factors such as state regulatory systems, the role of alcohol production and consumption in the state economy, and stakeholder influence. This analysis suggests that regional and cultural factors may warrant further consideration in targeted policy or health messaging interventions focused on excessive drinking, ensuring sensitivity to the region’s dominant culture. This is especially true since the patterns of excessive drinking across the *American Nations* regions appear to be heterogeneous. Despite this, the cultural identity of the region is likely to be very stable since a newcomer assimilates to the dominant culture of the region, which is a common anthropological observation. Messages may need to be tailored. For example, alcohol reduction messages in Greater Appalachia and the Deep South may emphasize a focus on self-determination and personal freedom (i.e., “protecting your health and freedom”). Whereas messages in Yankeedom and Left Coast focused on the “common good” or better health for the community. Certainly, these messages will need further exploration.

The previous work linking socioeconomic factors such as income, education, and employment status to excessive alcohol use should also be considered, especially given the previous literature linking these factors to alcohol consumption [29]. We are limited in this analysis by the cross sectional nature of the descriptive observation of excessive alcohol consumption patterns in the American Nation framework. Therefore, future work may focus on incorporating these measures. Furthermore, it is recognized that certain cultures and groups represented across the American Nations may have disparate health consequences as a result of excessive alcohol. This may be an important consideration for regions such as the El Norte and First Nation where Hispanic and Indigenous Americans are more populous. With these analysis, the *American Nations* model may be particularly helpful in crafting future alcohol reduction messages, interventions, and health policy recommendations.
